# Editorial: International day of yoga: understanding its neural mechanisms and clinical applications in brain health

**DOI:** 10.3389/fnhum.2025.1625827

**Published:** 2025-06-19

**Authors:** Cláudia Fetter, Maria Cláudia Irigoyen

**Affiliations:** ^1^Clinical Investigation Laboratory (LIC), Institute of Cardiology of Rio Grande do Sul, University Foundation of Cardiology (IC-FUC), Porto Alegre, Brazil; ^2^Experimental Laboratory of Hypertension, Heart Institute (InCor), University of São Paulo (USP), São Paulo, Brazil

**Keywords:** yoga, brain, neural mechanisms, cognitive function, acquired brain injuries (ABI)

## Introduction

Yoga has become popular in the West as a practice that helps enhance people's health and improve their physical and mental well-being (Sedlmeier and Srinivas). There is a growing scientific interest in investigating the benefits of yoga on human health with an increasing number of publications every year (Jeter et al., 2015). Most yoga studies have focused on *asana* (physical poses), *pranayama* (respiratory control) or meditation and these practices have been widely used to improve physiological outcomes (Fetter et al.), psychological states (Chaudhry et al., 2023) and brain health (Gothe et al., 2019).

Yet, yoga practices are complex and extrapolating results is a challenge compared to exercise studies. To deepen our understanding of the underlying neural mechanisms and clinical applications of yoga for enhancing brain health we often have to appraise the results from randomized controlled trials (RCT) with different methodological approaches and reporting weaknesses (Chobe et al., 2020). To deal with this problem, a large number of observational studies have recruited regular yoga practitioners and compared them to non-yoga groups. A systematic review of observational studies has showed that practicing yoga may have a significant impact on brain structure and function (Gothe et al., 2019).

Though neural mechanisms of yoga have been largely unexplored, its effects on the autonomic nervous system activity, by enhancing vagal modulation and promoting the “rest and digest” response, have been demonstrated to improve several different physical and mental functions (Frank et al., 2020). These findings from yoga studies are believed to be due to the effects of slow breathing (Deepeshwar and Budhi, 2022).

Voluntary slow breathing may have significant effects on physical, mental and psychological health, and is considered a low-cost, low-tech technique with few potential adverse effects (Laborde et al., 2022). To elucidate how slow breathing interacts with the human brain through multiple pathways, experimental studies have been conducted to determine the mechanisms involved, such as optogenetic activation of the corticopontine pathway in mice that slows breathing and alleviates behaviors associate with negative emotions (Jhang et al., 2024).

Despite advances in understanding the neural mechanisms involved in the widespread impact of yoga on brain health, dose-response effects of yoga components in different populations and their association with factors such as previous physical experience and cognitive ability need to be further explored (Datta et al., 2023).

## Highlights from this Research Topic

Stephens et al. has made an important contribution comparing in a pilot RCT the effects of group yoga and group low-impact exercise in adults with chronic acquired brain injuries presenting functional impairments such as poor balance and autonomic nervous system dysfunction. Group yoga and group exercise sessions were held twice a week for 1 h for 8 weeks led by trained adaptive exercise specialists. The primary outcomes were balance and autonomic nervous system function. They concluded that, due to its accessibility and holistic nature, yoga has significant potential for improving balance and autonomic nervous function, along with other capacities, in adults with chronic acquired brain injuries.

Kawazu et al. shed light on the efficacy and safety of mindfulness yoga intervention in children with school refusal often associated with anxiety and depression. They conducted a multicenter, exploratory, cluster RCT where the participants were randomly assigned to a non-yoga or a yoga group (a 4-week mindfulness yoga program through video sessions) and treatment as usual. The primary outcome was anxiety assessed using the Spence Children's Anxiety Scale (SCAS). They found that the mindfulness yoga program did not have a significant effect on anxiety compared with treatment as usual, but there was improvement in the Physical Injury Fears subscale and pulse rates were significantly lower in the yoga group compared to the non-yoga group.

Szaszkó et al. demonstrated the effects of yoga on executive control processes involving cognitive functions that are essential for everyday activities. They conducted an RCT including 98 participants where healthy yoga novices practiced Hatha yoga three times a week for eight weeks. These authors found that the intervention promoted increased task-related frontocentral theta activity, indicating an ability to increasingly deploy executive resources to the prioritized task when needed. They concluded that Hatha yoga has a positive influence on executive control.

Maity et al. reported that a prior history of poor health and unhealthy lifestyle choices among Indian prison inmates contributes to accelerated aging leading to cognitive impairment, which imposes economic and medical challenges to correctional health care systems. They argued that adapting yoga-based interventions for the implementation of well-structured rehabilitative programs can improve the lives of people with cognitive deficits in prison settings.

## Conclusions

This Research Topic of articles aims to present the current knowledge on the effects of yoga on neural mechanisms and its clinical applications to enhance brain health, as can be seen in the high-level featured publications, though fewer than expected in number. Further research is necessary to explore how yoga promotes neuroplasticity and may potentially enhance brain health.
